# Does wearing facemasks during small animal surgery reduce the incidence of surgical site infections?

**DOI:** 10.18849/ve.v9i4.690

**Published:** 2024-11-04

**Authors:** Alexander James Bartlett

**Keywords:** animal, facemask, infection, small, surgery, surgical, veterinary

## Abstract

**PICO question:**

In small animals undergoing theatre-based surgical procedures does having all theatre personnel masked, compared with not having all theatre personnel masked, result in a reduced incidence of surgical site infections?

**Clinical bottom line:**

**Category of research:**

Incidence.

**Number and type of study designs reviewed:**

Zero. No records relevant to the PICO question were retrieved from the literature search.

**Strength of evidence:**

Zero.

**Outcomes reported:**

There is no published evidence specific to small animals that assesses the effect of wearing facemasks during surgical procedures on the rate of postoperative surgical site infections.

**Conclusion:**

In small animals undergoing theatre-based surgical procedures, there is no published evidence evaluating the effect of having all theatre personnel masked compared with not having all theatre personnel masked on the incidence of surgical site infections. However, this lack of evidence should not be interpreted as lack of efficacy.

## How to apply this evidence in practice

The application of evidence into practice should take into account multiple factors, not limited to: individual clinical expertise, patient’s circumstances and owners’ values, country, location or clinic where you work, the individual case in front of you, the availability of therapies and resources.

Knowledge Summaries are a resource to help reinforce or inform decision-making. They do not override the responsibility or judgement of the practitioner to do what is best for the animal in their care.

## Clinical scenario

As clinical director of a veterinary hospital you are investigating strategies to improve the sustainability of your practice. You know from colleagues working at other practices that wearing facemasks during surgery is variable and wonder whether using fewer disposable surgical masks could help reduce the amount clinical waste produced. However, you worry that this could have a negative impact on the rate of post-operative surgical site infections.

## The evidence

There is no published evidence, specific to small animals, which assesses the effect of wearing facemasks during surgical procedures on the rate of post-operative surgical site infections. The literature search returned a total of 390 records. Of these, 388 were rejected from the title, one was rejected after assessing the abstract, and one was rejected as it was a congress proceeding. None of these records were relevant to the PICO question.

## Appraisal, application and reflection

The use of facemasks is considered best-practice for theatre attire in human surgery (National Institute for Care Excellence (NICE), 2013). This is both for the protection of the patient against surgical site infections (SSIs) originating from aerosolised infectious agents and for protection of the surgeons who are potentially at risk from fluid-borne infections (Davies et al., 2007; NICE, 2013). The evidence in human literature for facemasks reducing the incidence of surgical site infections is lacking. The most recent systematic review of randomised controlled trials assessing clinical outcomes on the subject concluded that there are too few papers of sufficient quality to confirm whether facemasks increase or decrease the risk of developing SSIs in human surgery (Burdick & Maibach, 2021). Another recent systematic review and meta-analysis which had wider inclusion criteria for human surgeries reported similar findings (Marson et al., 2020). With this lack of evidence, it has been questioned whether their mandatory use by all theatre personnel is relevant with modern aseptic practices (Da Zhou et al., 2015); however, the absence of evidence should not be mistaken for a lack of efficacy as there is indirect evidence that surgical mask wearing is protective against SSIs. These studies found that when surgeons did not wear masks the number of bacterial colonies formed on agar placed in various locations around the theatre increased compared to when surgeons were masked (Berger et al., 1993; Alwitry et al., 2002). This suggests that the risk of an SSI would be higher with unmasked theatre personnel, although the clinical significance of this indirect measurement is not known.

There are growing concerns over the sustainability of health care and a recent systematic review found that approximately 69% of the carbon footprint generated by products used during human surgeries came from single-use items and their disposal. Furthermore, disposable personal protective equipment, including disposable masks, contributed 11% of this total (Rizan et al., 2023). With the effectiveness of other single-use items being optimal for infection control starting to be questioned (Vasanthakumar, 2019; Bhutta, 2021), an evidence-based approach to the use of disposable surgical masks should be considered.

During the COVID-19 pandemic, the efficacy of facemasks was scrutinised. A shortage of personal protective equipment (PPE) forced theatre personnel to reuse surgical masks, where previously they were exclusively single-use items. The emerging evidence from retrospective studies comparing SSI incidences from surgeries performed pre-pandemic and during the pandemic suggests that extended use of surgical facemasks does not increase the risk of SSIs (Fraser et al., 2022; Malhotra et al., 2022). Therefore, despite a lack of evidence to justify complete removal of masks for theatre personnel, there is evidence to suggest their extended usage is safe. This would reduce the number of masks disposed of, reducing their environmental impact.

The quality of evidence from these studies does need to be considered as both are susceptible to bias and confounding factors. The inclusion criteria for Fraser et al. (2022) were limited to elective paediatric general surgery. This excluded emergency procedures and immunocompromised patients due to their perceived increased risk of SSIs. Consequently, it is difficult to generalise these results to adult patients, other types of surgery, or immunocompromised patients. The criteria used by Malhotra et al. (2022) were more inclusive, accounting for all surgical procedures excluding the first quarter of 2020 when the pandemic was being defined. The pre-pandemic and pandemic patient groups were also matched for uncontrolled variables using a propensity score algorithm. Their results show that despite extended use of surgical facemasks, hair coverings, and shoe coverings, there was a significant decrease in the SSI rate. This again suggests that extended use of single-use facemasks is safe.

Confounding factors associated with the pandemic were present in both studies. There was a public health campaign to increase vigilance of hand washing, there were fewer social interactions, and less physical contact between people. These factors may have contributed to improving general hygiene and consequently, a reduced risk of developing SSIs. Malhotra et al. (2022) acknowledged an increase in compliance of staff hand hygiene and increased use of N95 masks as opposed to standard surgical masks during the pandemic which may have concealed any negative effects on the SSI rate caused by extended use of face coverings.

In small animal veterinary medicine, facemask-use during surgery is variable between practices. The author has observed single-use surgical masks, reusable cloth masks, and no masks being worn by theatre personnel during surgery. This is likely due to a combination of tradition, individual practice policies, and an absence of a consensus regarding best practice. Research conducted in the veterinary environment would be more beneficial than extrapolating from human literature as the reduced resources available in veterinary surgery, such as a lack of positive pressure air control in theatres, may be significant. Inherent differences between the species may also introduce confounding variables. Future studies, ideally prospective randomised controlled trials with sub-category analysis for the type of surgery, are required to establish guidelines for a risk-based rather than blanket use of facemasks in surgery. Retrospective analysis of pre and post-pandemic data at other institutions would also be interesting to see if the trend of reduced SSI rates in humans with extended facemask usage during the pandemic are consistent.

Although there is a lack of evidence for the effectiveness of facemasks preventing SSIs during surgical procedures in human and veterinary surgery, the emerging data from the pandemic suggests that it may be possible to extend our use of disposable facemasks without detrimental effects on the rate of SSIs (Fraser et al., 2022; Malhotra et al., 2022). The benefits of this would be a reduction in the number of masks procured (Malhotra et al., 2022), an associated lower monetary cost (Bhutta, 2021), less waste generated from operating rooms (Vasanthakumar, 2019; Bhutta, 2021; Rizan et al., 2023) and allow these resources to be prioritised elsewhere for future pandemics. Even if facemasks were restricted to the scrubbed theatre personnel, as suggested by Webster et al. (2010), this could significantly reduce the amount of waste generated. Overinterpreting the absence of evidence as a lack of efficacy also needs to be avoided; however, the available evidence suggests that single-use mask policy may be unnecessary and other hygiene factors may be more important for reducing SSI rates than wearing masks during theatre-based procedures. Overall, the lack of conclusive clinical results and the presence of confounding factors currently make it impossible to recommend withdrawal of facemasks during small animal surgery.

## Methodology

**Search Strategy:** 

Databases searched and dates covered	CAB Abstracts on the OVID interface 1973 to 2023 Week 46 PubMed accessed via the NCBI website 1975 to 2023 Week 46
Search terms	CAB Abstracts: ('small animal*' or 'companion animal*') (dog or dogs or canis or canid* or canine* or bitch* or puppy or puppies).mp. or dog/ (cat or cats or felis or felid* or feline* or kitten or kittens or tom or toms or queen or queens).mp. or cat/ (Surg* or operat* or procedur*).mp. (mask or masks or facemask or facemasks or 'face cover*' or masked).mp. or mask/ (1 or 2 or 3) and 4 and 5 PubMed: “small animal*”[tiab] OR “companion animal*”[tiab] OR pets/ dog[tiab] OR dogs[tiab] OR canis[tiab] OR canid*[tiab] OR canine*[tiab] OR bitch*[tiab] OR puppy[tiab] OR puppies[tiab] OR dogs/ cat[tiab] OR cats[tiab] OR felis[tiab] OR felid[tiab] OR feline*[tiab] OR kitten*[tiab] OR tom[tiab] OR toms[tiab] OR queen[tiab] OR queens[tiab] OR cats/ mask[tiab] OR masks[tiab] OR facemask[tiab] OR facemasks[tiab] OR “face cover*”[tiab] OR masked[tiab] OR masks/ surg*[tiab] OR operat*[tiab] OR procedur*[tiab] (#1 OR #2 OR #3) AND #4 AND #5
Dates searches performed	27 Nov 2023

Table note placeholder

**Exclusion / inclusion criteria:** 

Exclusion	Not relevant to the PICO, human patients, not primary literature or review papers, non-English language.
Inclusion	Comparison of surgical site infection rates or proxy-measurements, small animal surgical procedures, direct data on theatre personnel wearing facemasks during procedures.

Table note placeholder

**Search Outcome:** 

Database	Number of results	Excluded – duplicated publications	Excluded – not relevant to the PICO	Excluded – not primary study or review paper	Total relevant papers
CAB Abstracts	226	1	224	1	0
PubMed	309	144	165	0	0
Total relevant papers when duplicates removed					0

Table note placeholder

## Acknowledgements

The author would like to gratefully acknowledge Clare Boulton (RCVS Knowledge) for her help with finalising the search strategy and conducting the database search.

## ORCiD

Alexander James Bartlett: https://orcid.org/0009-0008-0417-2391

## Conflict of interest

The author declares no conflicts of interest.

## Disclaimer

Knowledge Summaries are a peer-reviewed article type which aims to answer a clinical question based on the best available current evidence. It does not override the responsibility of the practitioner. Informed decisions should be made by considering such factors as individual clinical expertise and judgement along with patient’s circumstances and owners’ values. Knowledge Summaries are a resource to help inform and any opinions expressed within the Knowledge Summaries are the author’s own and do not necessarily reflect the view of the RCVS Knowledge. Authors are responsible for the accuracy of the content. While the Editor and Publisher believe that all content herein are in accord with current recommendations and practice at the time of publication, they accept no legal responsibility for any errors or omissions, and make no warranty, express or implied, with respect to material contained within. For further information please refer to our Terms of Use.
